# Cleavage of the Graft Bonds in PVDF–*g*–St Films by Boiling Xylene Extraction and the Determination of the Molecular Weight of the Graft Chains

**DOI:** 10.3390/polym11071098

**Published:** 2019-06-28

**Authors:** Jinhua Chen, Noriaki Seko

**Affiliations:** Department of Advanced Functional Materials Research, Takasaki Advanced Radiation Research Institute, National Institutes for Quantum and Radiological Science and Technology (QST), 1233 Watanuki-machi, Takasaki, Gunma 370-1292, Japan

**Keywords:** radiation grafting, graft chains, molecular weight, polystyrene, poly(vinylidene difluoride) (PVDF), pre-irradiation grafting

## Abstract

To determine the molecular weight of graft chains in grafted films, the polystyrene graft chains of PVDF–*g*–St films synthesized by a pre-irradiation graft method are cleaved and separated by boiling xylene extraction. The analysis of the extracted material and the residual films by FTIR, nuclear magnetic resonance (NMR), and gel permeation chromatography (GPC) analyses indicates that most graft chains are removed from the PVDF–*g*–St films within 72 h of extraction time. Furthermore, the molecular weight of the residual films decreases quickly within 8 h of extraction and then remains virtually unchanged up to 72 h after extraction time. The degradation is due to the cleavage of graft bonds, which is mainly driven by the thermal degradation and the swelling of graft chains in solution. This allows determination of the molecular weight of graft chains by GPC analysis of the extracted material. The results indicate that the PVDF–*g*–St prepared in this study has the structure where one or two graft chains hang from each PVDF backbone.

## 1. Introduction

In contrast to photo-induced and chemical reagent-induced graft polymerization, radiation-induced graft polymerization can introduce graft chains not only on the surface but also in the interior of solid materials, since the radiation can penetrate throughout the materials, thereby allowing a more uniform of radicals [1,2,3]. In general, the radiation grafting proceeds from the surface to the interior of the solid materials according to a diffusion-controlled grafting front mechanism [4,5]. The monomer used for radiation grafting can have opposite properties to the solid material. For example, a hydrophilic monomer can be grafted onto a hydrophobic backbone [6,7,8,9], and an ionic monomer can be grafted onto an insulating film [10,11,12]. Due to these versatile advantages, the radiation grafting has been used to modify existing films, fibers, and particles for applications such as ion-conductive membranes [13,14,15,16,17,18], adsorbents [19,20,21], sensor materials [9], and compatibilizers for polymer blends [22]. This radiation grafting is a “graft from” process in which free radicals that are produced on the backbones by irradiation initiated the polymerization of graft chains, forming C–C chemical bonds between graft chains and backbones, namely graft bonds [23,24,25].

Although the molecular weight of graft chains is an important parameter that affects the properties of grafted materials, it has rarely been studied [5,26,27]. By controlling grafting conditions, graft chains with designed molecular weight or length can be obtained for application in specific fields [28]. However, the difficult cleavage of the covalent graft bonds hinders the isolation of graft chains for analysis. For some special grafted materials using cellulose, chitosan, chitin, polyethylene oxide, or silica as the backbone, graft chains can be separated without degradation by chemical decomposition of the backbone [29,30,31,32]. Conversely, when chemically stable polyolefin or fluoropolymers are used as the backbone, graft chains are difficult to separate by the abovementioned decomposition method. With regard to the molecular weight, that of graft chains on the extreme surface of the solid materials is generally identical to that of the homopolymer because they are produced under similar conditions [4,26,27,33,34]. In contrast, the molecular weight of the inner graft chains is normally large than that of the homopolymer, since the former are produced via a solid reaction, whereas the latter results from a solution reaction [34,35,36].

Recently, the separation of materials chains from fluoropolymer-based grafted films has been achieved by immersion in hot water or solvents [17,18]. Nevertheless, in these cases, it is difficult to ascertain whether the separated materials correspond to graft chains or homopolymer. Similar observations were described in our previous study [35], in which chloromethylstyrene-grafted ethylene-tetrafluoroethylene copolymer (ETFE–*g*–CMS) films were immersed in xylene at 120 °C for more than 44 h. The ETFE–*g*–CMS films were prepared by a simultaneous radiation grafting method, from which a considerable amount of homopolymer was produced, and radiation-induced crosslinking occurred in the films. Therefore, it was difficult to confirm whether the obtained poly(CMS) stemmed from graft chains having chemical bonds with the ETFE backbones or from homopolymer interpenetrated into the ETFE backbones.

In this study, we aimed to investigate the cleavage of graft chains from grafted films using poly(vinylidene difluoride) (PVDF) as the backbones and polystyrene as graft chains, from which styrene-grafted PVDF (PVDF–*g*–St) films were prepared via a pre-irradiation grafting method [37]. Unlike the ETFE films, the PVDF films are soluble in polar aprotic solvents such as dimethylformamide (DMF) and N-methylpyrrolidone (NMP). Therefore, we expected that the PVDF-g-St films could be dissolved in these solvents, which would allow analysis of the molecular weight and to confirm graft bonds. Furthermore, by using the pre-irradiation grafting method, the formation of homopolymer within the PVDF films could be largely avoided.

Herein, we describe the successful separation of the polystyrene chains from the PVDF–*g*–St films by boiling xylene extraction. Furthermore, by characterizing the extracted material, the residual film, and the PVDF–*g*–St/NMP solution treated at the same temperature, a suitable method for the gel permeation chromatography (GPC) analysis of graft chains is developed, and the cleavage mechanism of graft chains from grafted films is investigated.

## 2. Materials and Methods

PVDF films with a thickness of about 20 μm were prepared by casting a DMF solution of PVDF powder (type, #1100, Kureha Chem. Co. Ltd., Tokyo, Japan) on a glass plate at 60 °C. The degree of crystallinity of the cast PVDF film was determined by differential scanning calorimetry (DSC 8230, Rigaku Corp., Tokyo, Japan) instrument, and was 34.3%. Styrene, methanol, xylene, NMP, DMF (GPC grade) and tetrahydrofuran (THF, GPC grade)) were purchased from Wako Pure Chemical Industries, Ltd. (Tokyo, Japan) and were used without any further treatment. Ultra-pure water was used throughout the work. All other materials and chemicals are analytical grade and were used as received.

**Scheme 1:**
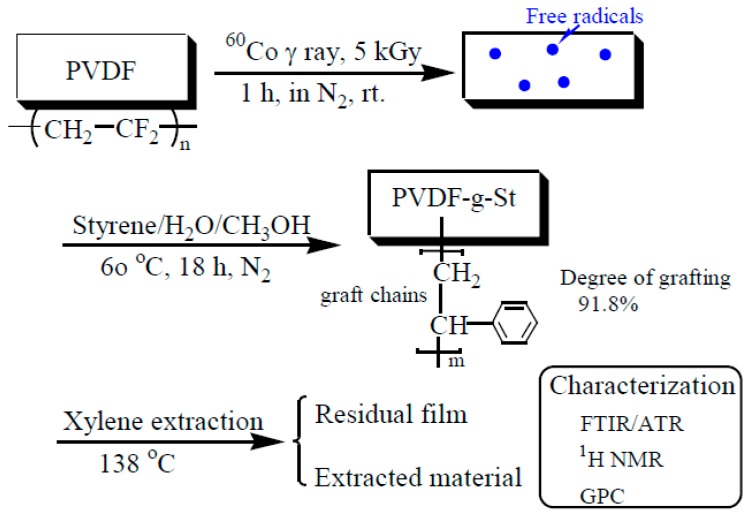
Process for the preparation and extraction of the PVDF–*g*–St film.

The process for the preparation and extraction of grafted film was presented in Scheme 1. The PVDF–*g*–St films were prepared using a pre-irradiation grafting method [37]. For this purpose, a special reaction tube containing the PVDF films (492.5 mg) was degassed for 1 h and then filled with nitrogen gas for pre-irradiation using the Co-60 gamma rays as irradiation source. The irradiation rate was 5.0 kGy h^−1^ and the total irradiated dose was 5.0 kGy. After the irradiation, a monomer solution consisting of 10 g of styrene, 20 g of water, and 40 g of methanol was injected into the reaction tube and then bubbled with nitrogen gas for 5 min to remove the oxygen. The graft polymerization was carried out by placing the reaction tube in an incubator shaker controlled at 60 °C for 18 h. After the graft polymerization, grafted films were taken out and then immersed in toluene in a glass bottle, which was shaken at 60 °C for 24 h to remove the homopolymers on the surface, and then dried in a vacuum oven at 60 °C for 24 h before use.

The degree of grafting of the resulting PVDF–*g*–St films was determined to be 91.8% using the equation: (*W*_g_ − *W*_o_)/*W*_o_ × 100%, where *W*_o_ and *W*_g_ are the weights of the films before and after grafting, respectively. This indicates that about 452 mg of the polystyrene chains were grafted into the PVDF backbones. The homopolymer formed in the monomer solution was collected by removing the monomer and the solvent by using a rotary evaporator at 60 °C for 5 h. About 570 mg of homopolymer formed in the monomer solution was collected, which suggests that most of the styrene was not polymerized and remained in the solution during the graft polymerization.

The extraction was carried out by immersing the PVDF–*g*–St film (about 10 mg) in xylene (10 g) and then refluxing at the boiling temperature of 138 °C. After the extraction, the residual film was taken out, washed with fresh xylene at room temperature and then weighed to obtain its wet weight. Then, the wet film was dried in a vacuum oven at 60 °C for 24 h to obtain its dry weight. The weight residue (%) of the film was calculated using the formula *W*_e_/*W*_g_ × 100, where *W_g_* and *W*_e_ are the dry weights before and after the extraction. The xylene uptake (%) of the film was calculated as (*W*_w_ − *W*_e_)/*W*_e_ × 100, where *W*_w_ is the wet weight of the film before drying. Meanwhile, the extraction solution was directly injected into the GPC instrument for molecular weight analysis.

The PVDF–*g*–St film, pristine PVDF, and commercial polystyrene were dissolved in NMP for thermal treatment at the same temperature (138 °C). During the thermal treatment, an aliquot of the solution was taken out and was injected into the GPC instrument for analysis.

The PVDF–*g*–St film, the residual film after xylene extraction, and the homopolymer obtained from the monomer solution were dissolved in DMF for GPC analysis. The extraction solution and thermally treated PVDF-g-St/NMP solution were directly used for GPC analysis. The GPC instrument (Hitachi High-Tech. Sci. Corp., Tokyo, Japan) was equipped with two columns (GPC KD-806M, Shodex Corp., Tokyo, Japan), a UV detector (270 nm, Hitachi High-Tech. Sci. Corp., Tokyo, Japan), and a refractive index (RI) detector (Hitachi High-Tech. Sci. Corp., Tokyo, Japan). The columns were controlled at 40 °C. DMF containing 0.1 wt.% of LiBr was used as the eluent and was flowed through the columns at a rate of 1.0 mL/min. The true molecular weight of the PVDF, especially that of the branched PVDF–*g*–St, was difficult to obtain. Instead, the molecular weights relative to the standard polystyrene were calculated [38].

The PVDF–*g*–St film, pristine PVDF, commercial polystyrene, and residual film with different extraction times were analyzed by Fourier transform infrared (FTIR) spectroscopy (PerkinElmer Japan Co., Ltd., Yokohama, Japan) in an attenuated total reflectance (ATR) mode. The FTIR spectra were recorded with a resolution of 4 cm^−1^ and 64 scans in the range from 500 to 3200 cm^−1^.

The starting PVDF–*g*–St film, 72 h-extracted residual film, and the corresponding extracted material were dissolved in deuterated DMF (DMF-7d, Wako Pure Chemical Industries, Ltd., Tokyo, Japan) for ^1^H nuclear magnetic resonance (NMR) analysis using a Bruker AV 300 MHz spectrometer (Bruker Corp., Yokohama, Japan). The extracted material was obtained by rotary evaporating the solvent from the corresponding extraction solution at 60 °C. About 0.5 mL of the samples was transferred to an NMR tube, and the ^1^H NMR spectra were recorded at room temperature.

## 3. Results and Discussion

### 3.1. Preparation of PVDF–g–St Films

The PVDF–*g*–St films were prepared by a pre-irradiation grafting method in which the PVDF films were pre-irradiated with gamma rays in nitrogen gas at room temperature [15,16]. During the irradiation, free radicals are produced on the PVDF chains due to the scission of chemical bonds such as C–H and C–F [1,2,3]. The free radicals in the PVDF films are stable at room temperature even for several days after the pre-irradiation [10,39]. The irradiated PVDF films were then immersed in a nitrogen gas-bubbled styrene solution at 60 °C to initiate the graft polymerization by the free radicals, which would afford polystyrene graft chains chemically bonded to the PVDF backbones. Both the area and thickness of the grafted films increased after the grafting. Moreover, the grafted films became translucent. The high degree of grafting of the prepared PVDF–*g*–St films (91.8%) suggests that the weight of the polystyrene graft chains and the PVDF backbones were almost equal. The PVDF–*g*–St film was then immersed in boing xylene for extraction. During the extraction, despite a significant swelling, the film remained its form and did not dissolve in the solvent. This is most likely because xylene is a good solvent for the polystyrene chains and a poor solvent for the PVDF backbones. Thus, two phases were formed in the PVDF–*g*–St film, namely a shrunken PVDF phase and a xylene-swollen polystyrene phase.

**Figure 1:**
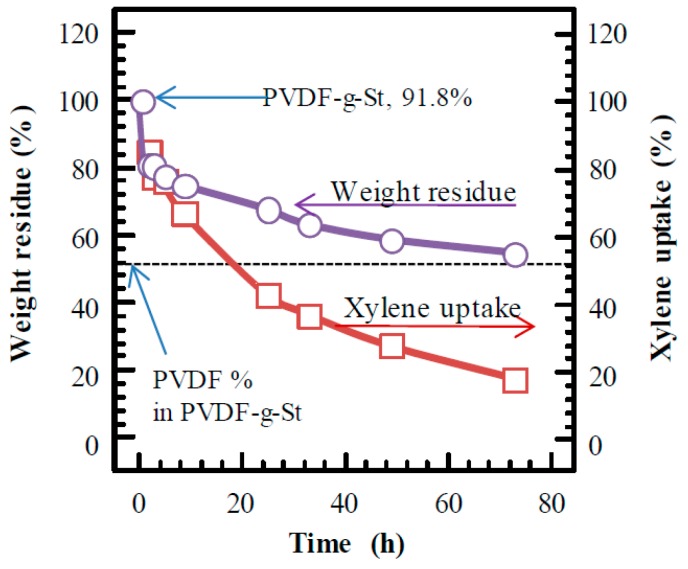
Weight residue and xylene uptake of the residual PVDF–*g*–St film (initial degree of grafting, 91.8%) under xylene extraction at 138 °C as a function of time. The dotted line means the weight percentage of PVDF backbone in the starting PVDF–*g*–St film, 52.0%.

Surprisingly, as shown in Figure 1, the weight of the residual film greatly decreased as the extraction proceeded. The weight of the film quickly dropped to 82% in the first hour and then slowly decreased to 55% at 72 h of extraction time. The starting PVDF–*g*–St film was translucent, whereas the 72 h-extracted residual film was transparent. In addition, the area and thickness of the 72 h-extracted residual film were significantly reduced, and its weight was close to that of the pristine PVDF film.

Furthermore, as can be also seen from Figure 1, the xylene uptake of the film decreased with increasing the extraction time because of the good affinity of the polystyrene in the film for xylene. This decrease in xylene uptake indicates the loss of polystyrene in the grafted film during the extraction. Even so, the xylene uptake remained fairly high after 72 h of extraction time, which may be due to the absorption of xylene in the pores formed during the extraction.

Several reasons can be invoked to explain the weight loss of the PVDF–*g*–St film during the extraction process: 1) dissolution of the homopolymer formed in the film; 2) cleavage of graft chains from the grafted film; and 3) thermal degradation of the grafted film. In our previous work [35], we prepared an ETFE–*g*–CMS film by a simultaneous irradiation grafting method and extracted the resulting film in xylene at 120 °C. We found that a considerable amount of poly(CMS) was extracted from the grafted film, which we attributed to the formation of homopolymer in the film. Indeed, the simultaneous irradiation grafting method can lead to the production of a large amount of homopolymer.

The pre-irradiation grafting method was selected in the present study to avoid homopolymerization, since free radicals are formed on the PVDF chains, ensuring that the graft chains are chemically bonded to the PVDF backbones. To verify that the graft chains were cleaved from the backbones under the extraction process, FTIR, NMR, and GPC analyses were performed on the extracted materials and the residual grafted films.

### 3.2. FTIR Characterization

**Figure 2:**
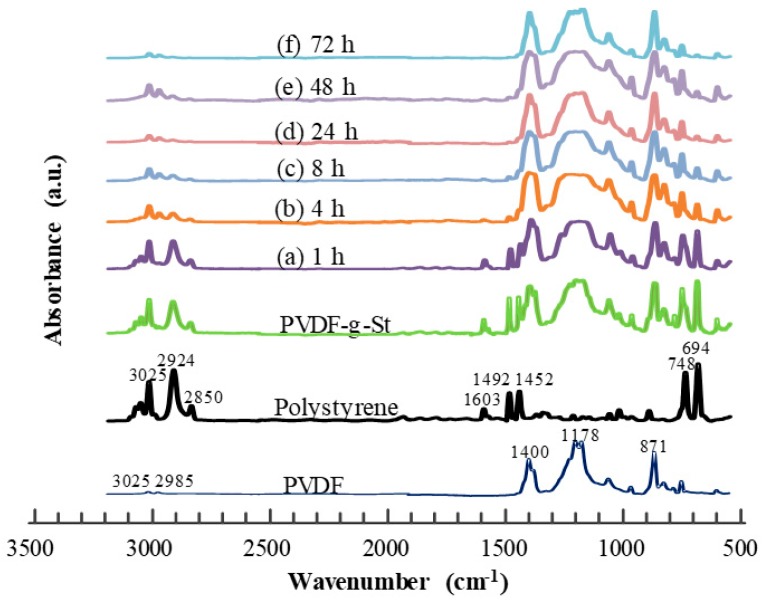
FTIR/ATR spectra of the residual PVDF–*g*–St film after xylene extraction with different times (a–f) together with those of the starting PVDF–*g*–St film, commercial polystyrene, and pristine PVDF film.

The extracted residual films and the starting grafted film were characterized by FTIR. For comparison, the pristine PVDF film and commercial polystyrene were also characterized under the same conditions. As shown in Figure 2, peaks at 3,025 cm^−1^ (CH_2_ asymmetric stretching), 2,985 cm^−1^ (CH_2_ symmetric stretching), 1,400 cm^−1^ (CH_2_ scissoring deformation), 1,178 cm^−1^ (CF_2_ asymmetric stretching), and 871 cm^−1^ (CH_2_ rocking vibration) appear in the PVDF spectrum. Meanwhile, that of polystyrene exhibits peaks at 2,924 cm^−1^ (CH_2_ asymmetric stretching), 2,850 cm^−1^ (CH_2_ symmetric stretching), and 1,452 cm^−1^ (CH deformation) that can be assigned to the CH_2_CH aliphatic chains, and peaks at 3,025 cm^−1^ (CH asymmetric stretching), 1,492 cm^−1^ (CH scissoring deformation), 1,603 cm^−1^ (C–C stretching), and 748 and 700 cm^−1^ (CH bending, mono-substituted) that are attributable to the aromatic groups. All the peaks for PVDF and polystyrene can be observed in the spectrum of PVDF–*g*–St, indicating that the polystyrene was implanted into the PVDF film.

As the extraction proceeds, as shown in Figure 2, the intensity of the peaks assigned to polystyrene, such as those at 2,924, 1,603, and 1,492 cm^−1^, decreases, whereas the peaks of PVDF (1,400, 1,178 and 871 cm^−1^) remain virtually unaltered. The spectrum of the 72 h-extracted residual film was found to be similar to that of the pristine PVDF film. Therefore, it seems reasonable to conclude that the polystyrene chains in the film, whether or not grafted to the PVDF backbones, were continuously removed from the grafted film under xylene extraction.

### 3.3. NMR Characterization

**Figure 3:**
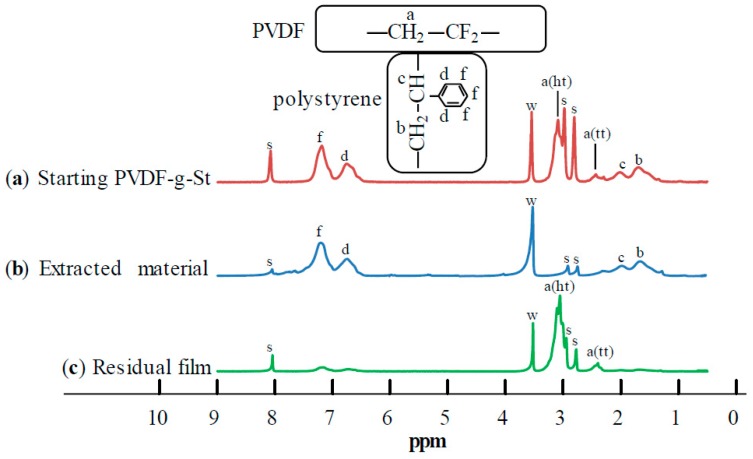
^1^H NMR of (**a**) starting PVDF–*g*–St film; (**b**) extracted material; and (**c**) 72 h-extracted residual film.

The 72 h-extracted residual film, the corresponding extracted material, and the starting PVDF–*g*–St film were examined by NMR spectroscopy. As shown in Figure 3a, the spectrum of the starting PVDF–*g*–St film shows peaks around 6.5–7.5 and 1.3–2.6 ppm, which can be attributed to the aromatic protons and the protons of the CH_2_CH aliphatic groups of polystyrene, respectively. In addition, peaks at 3.10 and 2.43 ppm can be assigned to the head-to-tail and head-to-head configurations of PVDF [40,41,42]. The other peaks at 2.74, 2.91, 8.01 and 3.00 ppm are due to trace amounts of DMF and water. For the extracted material obtained from the extraction solution, as shown in Figure 3b, only the peaks assigned to polystyrene and to the impurities are observed. Therefore, it can be concluded that polystyrene was extracted from the grafted film, whereas the PVDF remained in the film during the extraction.

In contrast, Figure 3c shows that the peaks for the polystyrene almost disappear in the spectrum of the 72 h-extracted residual film, whereas those for PVDF are present. These results prove that the weight loss of the grafted film during the extraction is due to the dissolution of polystyrene into the solvent. Furthermore, most of the polystyrene, with or without graft bonds, was removed from grafted film under the xylene extraction, indicating that the polystyrene and PVDF could be well separated. These results agree with those of the FTIR analysis.

### 3.4. Degree of Separation and Grafting of the Residual Grafted Film

**Figure 4:**
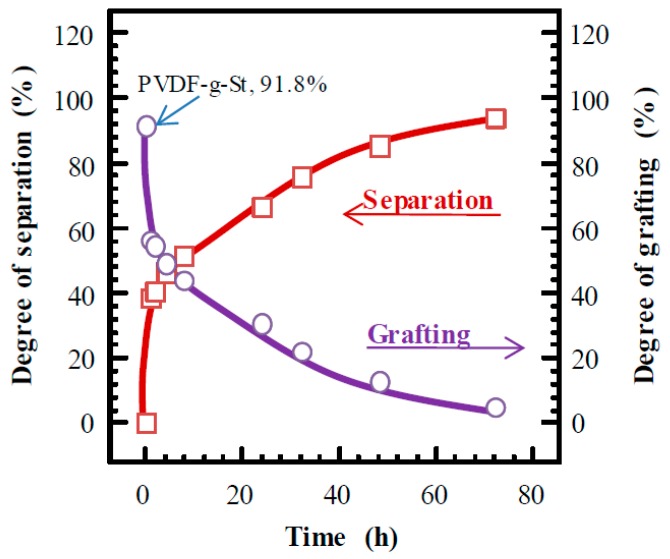
Degree of separation and degree of grafting of the residual PVDF–*g*–St film (initial degree of grafting, 91.8%) in xylene at 138 °C as a function of extraction time.

Based on the FTIR and NMR results, we conform that the polystyrene can be separated from grafted film. Figure 4 shows the degree of separation and grafting of the residual grafted film as a function of extraction time. The degree of separation was calculated as the percentage of polystyrene weight loss in the grafted film relative to the total weight of polystyrene in the starting grafted film, using the equation (*W*_g_ − *W*_gt_)/(*W*_g_ − *W*_o_) × 100, where *W*_o_ is the weight of the pristine PVDF film, *W*_g_ is the weight of the starting grafted film, and *W*_gt_ is the weight of the residual grafted film. As shown in Figure 4, the degree of separation increased sharply to about 46% in the initial 4 h and then slowly increased to reach 94% at 72 h of extraction time. Therefore, most of the polystyrene was removed from the grafted film and dissolved in the xylene solvent after 72 h of extraction time.

In contrast, due to the loss of polystyrene, the degree of grafting decreased during the extraction. As shown in Figure 4, the degree of grafting dropped sharply from 91.8 to 49.2% in the initial 4 h and then slowly decreased to 5.5% after 72 h of extraction time as the polystyrene was removed from the grafted film.

These results confirm that PVDF and polystyrene in the PVDF–*g*–St film were well separated by boiling xylene extraction. Nevertheless, the extreme case in which the extracted material was homopolymer and the remained polystyrene chains in the film after extraction were the real graft chain cannot be dismissed at this stage. In that case, the abovementioned 91.8% and 5.5% values would correspond to the apparent degree of grafting and the real degree of grafting of the PVDF–*g*–St film, respectively. To rule out such extreme case and to determine whether the separation is due to the cleavage of the graft bonds or the degradation of the polystyrene chains, we analyzed the molecular weight and thermal stability of the grafted film, the extracted material, and the residual film.

### 3.5. GPC Analysis of the Residual Grafted Films

**Figure 5:**
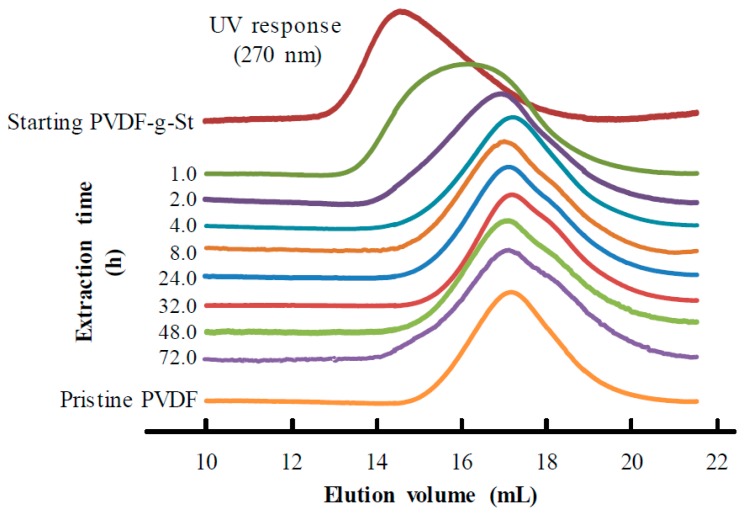
GPC traces of the residual PVDF–*g*–St films with different extraction times, corresponding starting PVDF–*g*–St film, and pristine PVDF film. Eluent, 10 mM LiBr in DMF; flow rate, 1.0 mL/min; column temperature, 40 °C; UV detector, 270 nm.

Figure 5 shows the GPC traces of the residual grafted films, the starting grafted film, and the pristine PVDF film recorded using the UV response (270 nm) detection mode. As can be seen in the figure, all the UV responses of the samples showed a single positive peak, which shifted to high elution volume as the extraction time increased. The peaks corresponding to homopolymer and ungrafted PVDF were not observed in the UV response mode.

**Figure 6:**
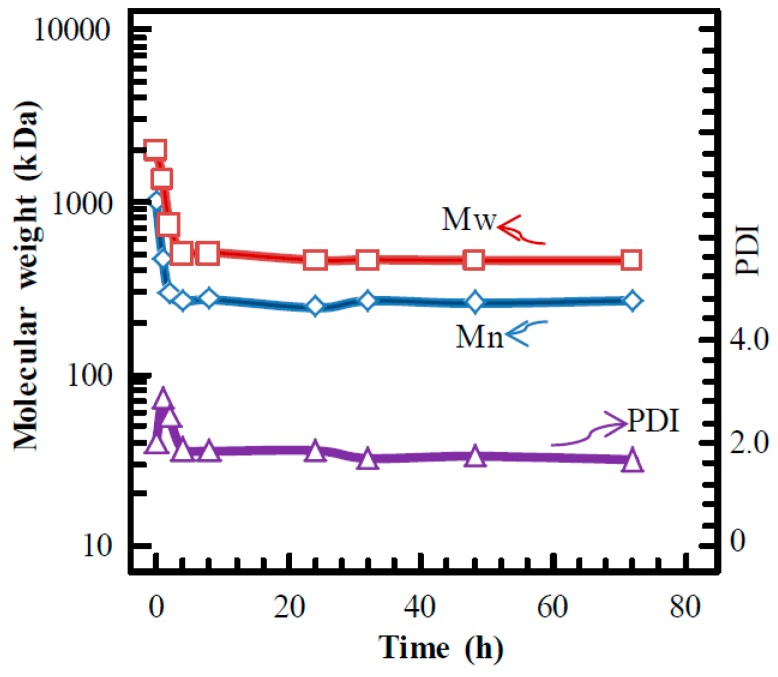
Molecular weight (*M*_w_ and *M*_n_) and polydispersity index (PDI) of the residual PVDF–*g*–St films with different extraction times. The data were calculated from Figure 5 using standard polystyrene for calibration curve. Eluent, 10 mM LiBr in DMF; flow rate, 1.0 mL/min; column temperature, 40 °C; UV detector (270 nm).

The molecular weight and polydispersity index (PDI) of the samples were calculated from Figure 5 using standard polystyrene for the calibration curve. The *M*_n_ and *M*_w_ values of the pristine PVDF film were determined to be 284 and 460 kDa, respectively. In contrast, *M*_n_ an*d M*_w_ of the PVDF–*g*–St film were 996 and 1,979 kDa, respectively. This large increase in molecular weight of grafted film indicates that the polystyrene chains were grafted to the PVDF backbones through chemical bonds [43], which enable us to rule out the abovementioned case of a large amount of homopolymer being produced in the film. As shown in Figure 6, the molecular weight of the residual grafted film decreased as the extraction proceeded. This decrease was significant during the initial 8 h of extraction time, which indicates that the grafted film was quickly degraded. The residual film could be composed of polystyrene, PVDF, PVDF–*g*–St, and their segments; however, the molecular weight of the residual film remained almost unchanged in the extraction region of 8–72 h. On the other hand, the weight of the residual film was found to decrease continuously up to 72 h of extraction time (see Figure 1). Therefore, its degradation is most likely due to the scission of the graft bonds within the first 8 h of extraction time. After that, the residual film would be mainly composed of PVDF and polystyrene. Both were relatively stable and hardly degraded under the xylene extraction conditions, and hence the molecular weight was almost unchanged from 8 to 72 h of extraction time. Although most of the graft bonds were cleaved within 8 h, the weight of the residual film decreased continuously up to 72 h of extraction time, indicating that the weight loss of the residual film was a polystyrene diffusion-controlling process.

The residual film exhibited the highest PDI of 2.85 at 1 h of extraction time. The 1 h-extracted residual film was composed of PVDF–*g*–St, polystyrene, and PVDF. Since the molecular weight of PVDF–*g*–St was largely higher than those of polystyrene and PVDF, the molecular weight distribution of the mixture was very broad. After 8 h of extraction time, most of the graft bonds of PVDF–*g*–St were cleaved and thus the PDI remained almost constant.

**Figure 7:**
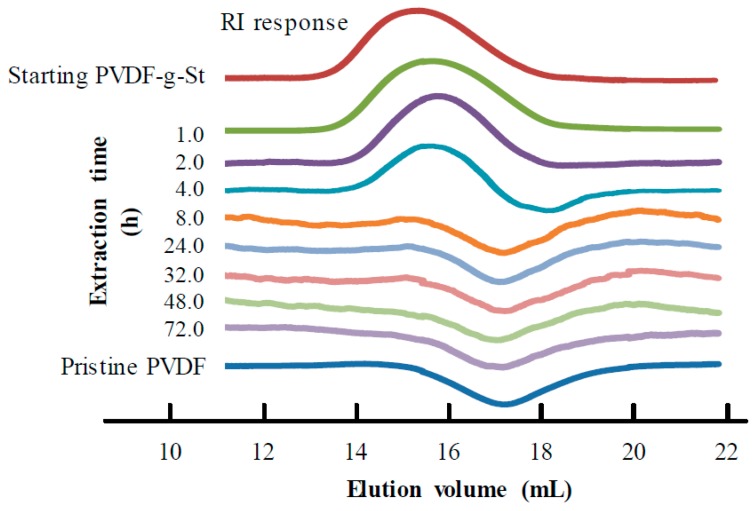
GPC traces of the residual PVDF–*g*–St films with different extraction times, corresponding starting PVDF–*g*–St film, and pristine PVDF film. Eluent, 10 mM LiBr in DMF; flow rate, 1.0 mL/min; column temperature, 40 °C; RI detector.

In contrast, as shown in Figure 7, the GPC traces in the refractive index (RI) response mode exhibited a relatively complex curve with positive and negative peaks. This is because the refractive index of DMF is lower than that of polystyrene but higher than that of PVDF [44,45]. Thus, the negative peak for PVDF can be observed on the GPC traces in the RI response mode. The starting PVDF–*g*–St film and the pristine PVDF film gave rise to a single positive and negative peak, respectively. During the xylene extraction, the positive peak shifted to high elution volume, and a negative peak appeared. The occurrence of the negative peak indicates that PVDF backbones without polystyrene graft chains appeared in the residual film as the extraction proceeded. After 8 h of extraction the positive peak almost disappeared. Moreover, the 72 h-extracted residual film afforded a GPC trace similar to that of the pristine PVDF film. These results prove that the polystyrene was cleaved and separated from the grafted film, whereas PVDF remained in the film.

Due to the opposite response of the PVDF and polystyrene chains to the RI detector, the relative molecular weights of the residual film are difficult to obtain using the conventional calibration method; however, the information obtained from these experiments shed some light on the cleaving of the graft bonds during the extraction.

### 3.6. GPC Analysis of the Extracted Materials

**Figure 8:**
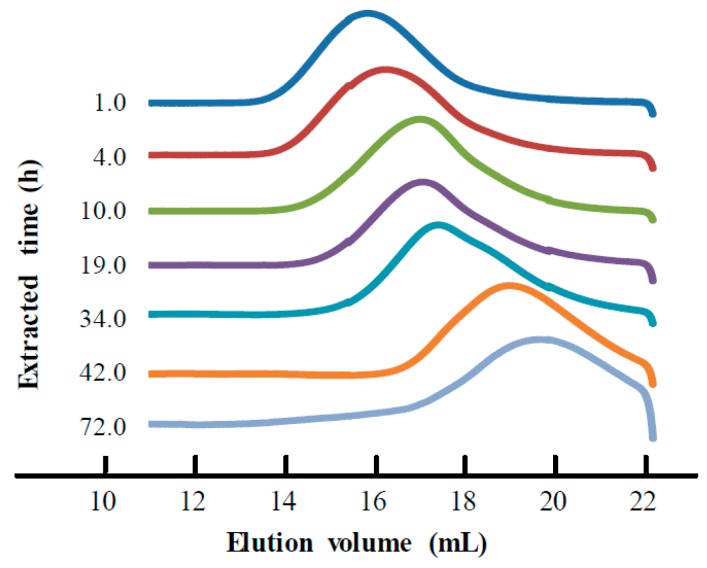
GPC traces of the extracted polystyrene graft chains obtained with different extraction times. GPC conditions: eluent, 10 mM LiBr in DMF; flow rate, 1.0 mL/min; column temperature, 40 °C; RI detector.

After confirming that the extracted materials were composed of polystyrene graft chains by NMR spectroscopy, the extraction solutions were directly injected into the GPC instrument. The results depicted in Figure 8 show that the peak shifted to high elution volume as the extraction proceeded, indicating that the polystyrene degraded slowly under the extraction conditions (the slow degradation of polystyrene cannot be detected in Figure 6 most likely due to the effect of the coexistence of PVDF).

**Figure 9:**
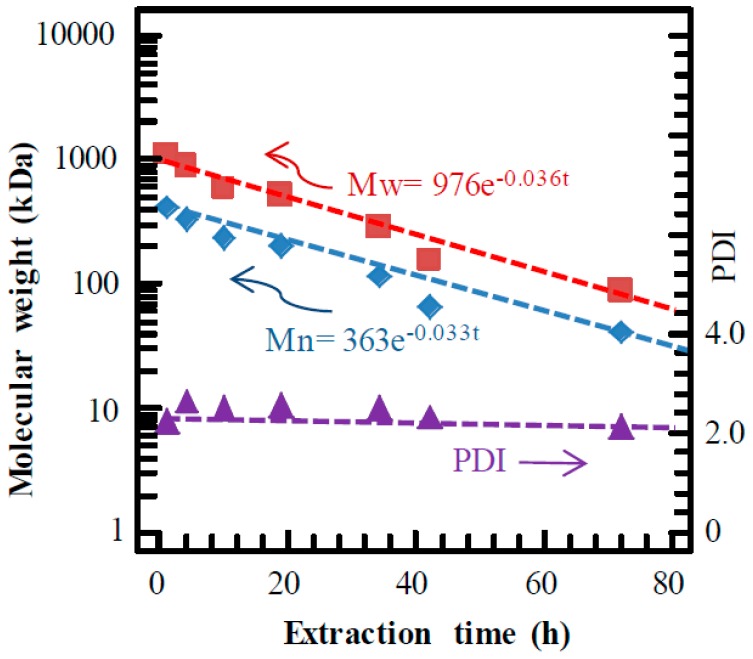
Relative molecular weight (*M*_w_ and *M*_n_) and polydispersity index (PDI) of the extracted polystyrene graft chains calculated from Figure 8.

Figure 9 displays the molecular weight (*M*_n_ and *M*_w_) and PDI (*M*_w_/*M*_n_) of the polystyrene obtained from Figure 8 as a function of extraction time. As can be seen, the molecular weight of polystyrene decreased with increasing extraction time, whereas the PDI remained virtually unchanged. Such an exponential decrease tendency can be attributed to the thermal degradation of the polystyrene in the xylene solution at high temperature [46]. From Figure 9, we can obtain the molecular weight of the graft chains without deterioration by extending the trend straight line to the extraction time of 0 h. Thus, the *M*_n_ and *M*_w_ of the non-degraded graft chains were determined to be 363 and 976 kDa, respectively. From the comparison with the *M*_n_ and *M*_w_ of the graft chains at 19 h extraction time (201 and 515 kDa, respectively), it can be concluded that about one C–C bond for each polystyrene chain was cleaved within 19 h of extraction time. Furthermore, within 4 h of extraction time, the *M*_w_ decreased about 8.9% (from 976 to 889 kDa) while the degree of separation of the graft chains reached more than 46% (see Figure 4). Therefore, the weight loss of the residual film during the boiling xylene extraction was mainly due to the cleavage of the graft bonds. As shown in Figure 6, most of the graft bonds were cleaved within 8 h of extraction time, which indicates that the cleavage of graft bonds was significantly faster than the degradation of the graft chains. Thus, most of the graft chains were integrally cleaved from the graft sites without degradation during the initial extraction period, thereby allowing the determination of the molecular weight of the graft chains.

The molecular weights of the graft chains are presented in Table 1. For comparison, the data for the pristine PVDF film, the starting PVDF–*g*–St film, and the polystyrene homopolymer obtained by rotary evaporating the monomer solution after the graft polymerization are also summarized. As can be seen in Table 1, the *M*_n_ and *M*_w_ of the pristine PVDF film were 284 and 460 kDa, respectively. After graft polymerization, the *M*_n_ and *M*_w_ of PVDF–*g*–St significantly increased to 996 and 1,979 kDa, respectively. On the other hand, the *M*_n_ and *M*_w_ of the polystyrene graft chains were 363 and 976 kDa, respectively. Therefore, the molecular weight of the polystyrene graft chains is of the same order of magnitude as that of the PVDF backbones, and each PVDF–*g*–St chain can be envisioned as one or two polystyrene graft chains hanging from the PVDF backbone, forming a T- or TT-type structure.

It should be noted that the molecular weight of the homopolymer was lower than that of the polystyrene graft chains. Similar results were found in our previous study [35], in which chloromethylstyrene was grafted onto an ETFE film by a simultaneous irradiation graft method. In the present study, graft polymerization was carried out with a pre-irradiated PVDF film, which, unlike the simultaneous irradiation graft method, considerably suppressed the homopolymerization. The slight homopolymerization is mainly attributed to the thermal polymerization. The propagating radicals in the monomer solution are more easily transferred or terminated, resulting in the relatively lower molecular weight of the homopolymer compared to that of graft chains.

### 3.7. Cleavage Mechanism

In our previous study [35], we extracted the ETFE–*g*–CMS film in xylene at 120 °C, finding that a considerable amount of poly(CMS) was separated and dissolved in the solvent. However, the nature of the extracted material, i.e., whether it was composed of graft chains or homopolymer, could not be ascertained because much homopolymer was produced in the ETFE–*g*–CMS film prepared by the simultaneous irradiation grafting method.

The method used in the present study circumvents considerably the formation of homopolymer. Furthermore, the FTIR and NMR analysis confirmed that graft bonds were cleaved and graft chains were separated, remaining the pristine PVDF in the solvent after the long extraction time.

**Figure 10:**
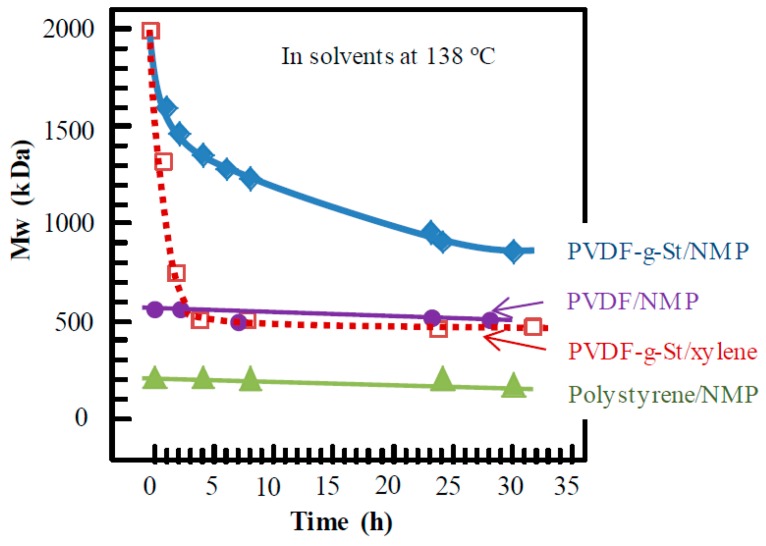
Weight-average molecular weight (*M*_w_) of the PVDF-*g*-St film, pristine PVDF and commercial polystyrene in NMP (bond symbols and lines) and the residual PVDF–*g*–St film in xylene (bulk symbols and dotted line) at 138 °C as a function of treatment time.

Next, we investigated the thermal stability of the PVDF–*g*–St film, pristine PVDF, and commercial polystyrene in NMP at 138 °C. In this case, the three polymers dissolved well in the NMP solvent. As shown in Figure 10, the PVDF–*g*–St sample was unstable in NMP, and its *M*_w_ decreased sharply from 1,979 to 1,240 kDa during the initial 8 h and then slowly dropped to 863 kDa at 30 h of treatment time. In contrast, PVDF and polystyrene were relatively stable, and their molecular weights decreased slowly during the thermal treatment in NMP. These results confirm that the thermal degradation of PVDF–*g*–St is due to the cleavage of graft bonds. Specifically, in NMP at 138 °C, the C–C chemical bonds in PVDF and polystyrene were stable, whereas those between PVDF and polystyrene, namely graft bonds, were weak, which resulted in their cleavage and the concomitant decrease in the molecular weight of grafted material. It is worth noting that the higher stability of polystyrene in NMP than in xylene at 138 °C may be because the latter is a better solvent for polystyrene, resulting in its faster degradation in xylene.

For comparison, the *M*_w_ of the residue PVDF–*g*–St film after xylene extraction is also presented in Figure 10. As expected, the molecular weight of the film decreased at a lower rate in NMP than in xylene indicating that the cleavage of graft bonds in xylene solvent was faster than in NMP.

**Figure 11:**
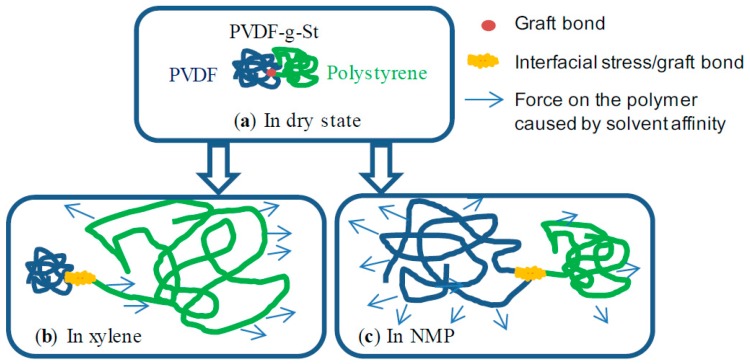
Cleavage mechanism of PVDF–*g*–St in dry state (**a**) and in solvents of xylene (**b**) and NMP (**c**). In xylene, the polystyrene chains tend to dissolve in solution while the PVDF chains tend to shrink. In NMP both the polystyrene and PVDF chains dissolve in solution but NMP are better solvent for PVDF chains than for polystyrene chains. The arrows refer to the force caused by interaction of the polymer chains and solvent molecules.

Figure 11 is presented to describe the cleavage phenomena of graft chains and the different behavior of PVDF–*g*–St in xylene and NMP. As shown in Figure 11, the PVDF–*g*–St chains in the dry state (a) were shrunken, and the PVDF and polystyrene chains were connected by graft bonds. The nonpolar PVDF and polar polystyrene chains did not interpenetrate with each other, affording distinct PVDF and polystyrene phases. However, in xylene (b), the PVDF–*g*–St film was not dissolved, whereas the polystyrene graft chains were swollen and the PVDF chains tended to shrink. This is because xylene is a poor solvent for PVDF but a good solvent for polystyrene. The thermal treatment induced the degradation of grafted film, and the opposite swelling behavior of the long PVDF and polystyrene chains enhanced the cleavage of graft bonds [47]. Similar phenomena regarding C–C scission induced by adsorption have been reported, where the steric repulsion between short graft chains promotes the breaking of the backbones [48,49]. The thermal treatment and the weak graft bonds are other reasons for the cleavage. The cleavage in xylene was a solid reaction.

On the other hand, in NMP (c), the solubility parameters of NMP, PVDF, and polystyrene were about 23.1, 23.2 and 18.0 (MPa)^0.5^, respectively. Therefore, PVDF tended to expand more expand than polystyrene, resulting in the interfacial stress on their joints. Furthermore, the low affinity between the PVDF backbones and the polystyrene graft chains in NMP accelerated their separation. Nevertheless, such interfacial stress was lower than in xylene. Even so, graft bonds were cleaved in NMP at 138 °C. The cleavage in NMP was a solution reaction.

In both cases, the interfacial stress was accumulated at the joints of the two components, i.e., graft chains and backbones. Overall, the interfacial stress, the thermal treatment, and the weak graft bonds cause the separation of graft chains from the backbones. However, the separated graft chains can be directly analyzed by using the xylene extraction, whereas graft chains and backbones coexist in NMP solution, and additional separation processing is required for the analysis of graft chains.

## 4. Conclusions

PVDF–*g*–St film with a high degree of grafting of 91.8% was prepared by a pre-irradiation grafting method. The polystyrene graft chains of a PVDF–*g*–St film were successfully cleaved and separated by boiling xylene extraction. The results of the FTIR, NMR, and GPC analyses confirmed that the polystyrene chains were dissolved into xylene, whereas the PVDF backbones remained in the film during the extraction. The cleavage of graft bonds was mainly due to the thermal degradation and was enhanced by the different swelling behavior of graft chains and the backbones. Thus, the molecular weight of graft chains of grafted film was successfully detected by cleaving graft bonds in boiling xylene and subsequent GPC analysis of the extracted material. The molecular weight of graft chains was of the same order of magnitude as that of the PVDF backbones and lower than that of the corresponding PVDF–*g*–St film. The PVDF–*g*–St chains have the structure as one or two polystyrene chains hanging from each PVDF backbone, forming a T- or TT-type structure.

## Figures and Tables

**Table 1:** Molecular weights of the pristine PVDF, starting PVDF–*g*–St, polystyrene graft chains, and the homopolymer.

Materials*	*M*_n_ (kDa)	*M*_w_ (kDa)	PDI (*M*_w_/*M*_n_)
Pristine PVDF	284	460	1.62
Starting PVDF–*g*–St film	996	1,979	1.99
Polystyrene graft chains	363	976	2.69
Polystyrene homopolymer	250	487	1.95

*The pristine PVDF is the film used for grafting, the starting PVDF–*g*–St-film has a degree of grafting of 91.8%, and the polystyrene homopolymer was the material obtained from the grafting solution.
